# Graph-Based Feature Weight Optimisation and Classification of Continuous Seismic Sensor Array Recordings

**DOI:** 10.3390/s23010243

**Published:** 2022-12-26

**Authors:** Jiangfeng Li, Lina Stankovic, Vladimir Stankovic, Stella Pytharouli, Cheng Yang, Qingjiang Shi

**Affiliations:** 1 Department of Electronic and Electrical Engineering, University of Strathclyde, Glasgow G1 1XW, UK; 2 Department of Civil and Environmental Engineering, University of Strathclyde, Glasgow G1 1XJ, UK; 3 College of Electronics and Information Engineering, Shanghai University of Electric Power, Shanghai 200090, China; 4 School of Software Engineering, Tongji University, Shanghai 201804, China; 5 Shenzhen Research Institute of Big Data, Shenzhen 518000, China

**Keywords:** feature engineering, multi-channel seismic events detection, graph feature weight optimisation and classification

## Abstract

Slope instabilities caused by heavy rainfall, man-made activity or earthquakes can be characterised by seismic events. To minimise mortality and infrastructure damage, a good understanding of seismic signal properties characterising slope failures is therefore crucial to classify seismic events recorded from continuous recordings effectively. However, there are limited contributions towards understanding the importance of feature selection for the classification of seismic signals from continuous noisy recordings from multiple channels/sensors. This paper first proposes a novel multi-channel event-detection scheme based on Neyman–Pearson lemma and Multi-channel Coherency Migration (MCM) on the stacked signal across multi-channels. Furthermore, this paper adapts graph-based feature weight optimisation as feature selection, exploiting the signal’s physical characteristics, to improve signal classification. Specifically, we alternatively optimise the feature weight and classification label with graph smoothness and semidefinite programming (SDP). Experimental results show that with expert interpretation, compared with the conventional short-time average/long-time average (STA/LTA) detection approach, our detection method identified 614 more seismic events in five days. Furthermore, feature selection, especially via graph-based feature weight optimisation, provides more focused feature sets with less than half of the original number of features, at the same time enhancing the classification performance; for example, with feature selection, the Graph Laplacian Regularisation classifier (GLR) raised the rockfall and slide quake sensitivities to 92% and 88% from 89% and 85%, respectively.

## 1. Introduction

Slope instabilities and landslides caused by meltwater drainage or heavy rainfall, as a result of climate change, is an increasing threat, especially around train tracks, main roads and densely populated mountain areas [1]. Progressive damage of the rocky slope is a precursor of slope instability, characterised by seismic events triggered by a rapid release of energy. Therefore, continuous seismic monitoring through an array of surface or near surface sensors, together with seismic analysis, comprising detection, classification, and localisation, has been gaining traction recently, whether events are induced by volcanic activity [2], landslides [3] or mining [4].

Many signal-processing and machine-learning approaches have been used for various seismic signal analysis tasks. Deep learning (DL) has recently become a popular technique in seismology. Indeed, many DL techniques based on Convolutional Neural Network [5], Deep Recurrent Neural Network [6], Capsule Neural Network [7] and Autoencoder [8] have been proposed to detect and classify earthquakes, as well as other seismic signals triggered by volcanoes and landslides. Though the aforementioned techniques can extract important features from the input data for detection and classification, it is not clear how this extraction is performed or how effective the extracted features are. Therefore, DL approaches are considered black-box models, which lack interpretability. Furthermore, in contrast to automatic feature learning offered by DL, handcrafted feature engineering can potentially lead to new insights and improved understanding of seismic signal patterns in temporal, spatial and frequency domains. Therefore, this work focuses on handcrafted feature engineering paired with traditional interpretable classification algorithms for seismic signals at an ongoing landslide site.

In seismic analysis, efficient and highly accurate seismic detection plays a crucial role. This is often accomplished by manually detecting signals of interest from continuous seismic recordings using expert knowledge, which is a time-consuming, laborious, and subjective process. As a result, algorithms that can automatically detect seismic signals have been developed. STA/LTA is a commonly used approach, but it suffers from inaccurate initialisation of parameters and leads to several false alarms [9]. Akaike information criterion (AIC), template matching, and thresholding algorithms are also used to detect seismic signals [9]. A one-step detection and classification approach is proposed in [3] to detect and classify seismic events by first sliding a window of predefined length on a continuous data stream from seismic sensor arrays, constructing 55 handcrafted features from each window to classify the window with Random Forest (RF) classifier. However, this one-step approach suffers from cumbersome feature construction, which is unsuitable for real-time applications. Additionally, the significance of the features associated with the whole waveform of the signal is not analysed because [3] only used the portions of the event’s waveform that appear in the sliding window. Since, the main challenge for seismic signal detection is very low signal-to-noise ratio (SNR) of the recorded signals and varying noise intensity and distribution [3,10], to enhance the quality of recorded multi-channel seismic signals, previous works employed stacked absolute values across different channels or the absolute value of the product of the amplitudes from different channels as the input for detection approaches. But the performance is still affected by the presence of strong noise in any of the recording channels [10]. To address this issue, this paper adapts the Multi-channel Coherency Migration (MCM) together with Neyman–Pearson lemma on landslide-induced signal detection and obtains convincing results even for the signals with small amplitudes.

With the detected signals from continuous multi-channel recordings, it is necessary to develop interpretable and effective feature engineering for event classification. The main challenges in classifying seismic signals are: (1) lack of open access annotated datasets [11]; (2) imbalanced catalog of labeled events, caused by the sparsity of events of interest [11]; (3) high similarities between unknown natural and anthropogenic “interfering” signals and events of interest in time and/or frequency domain [12]. Feature engineering is a key step towards efficient signal classification as a large set of features with redundant information could easily increase the processing time and cause classifier overfitting, multicollinearity, and suboptimal feature ranking at the selection stage [2]. Feature construction for seismic events was discussed in detail in [11], where temporal, spectral and cepstral features and combinations thereof are derived from the raw denoised measurements. Feature extraction [4] and selection [2] are commonly used for dimensionality reduction of the feature space, thus decreasing required storage requirements, and testing and training times of the classifier.

As the most popular feature extraction (and dimensionality reduction) method, Principal Component Analysis (PCA) has been consistently shown to be effective for a range of seismic detection and classification tasks, e.g., in [4] for microseismic event and quarry blast classification with Artificial Neural Networks (ANN), and [13] observed that PCA extracted features resulted in better classification accuracy for seismic events with linear and Radial basis function kernel Support Vector Machine (SVM) classifiers (like the one used for benchmarking in this paper). Although PCA is hampered by the high computational complexity of singular value decomposition, further calculated principle components cannot reliably identify the variables that are most crucial for information preservation and interpretability.

Feature selection methods are often categorised as filter-based (most popular for seismic analysis), wrapper-based, embedded, hybrid, and ensemble approaches. Filter-based methods, based on evaluating and selecting the features with various statistical tests, are model-agnostic, i.e., they can be applied to any learning algorithm to exclude irrelevant and redundant features, and are of lower complexity [14]. Thus, these methods have been widely used in various seismic analyses. For example, (i) [2] used Information Gain, One Rule, Relief, Chi2 Discretization, and uFilter filter feature selection approaches with a Gaussian Mixture Model classifier to classify volcano-seismic signals, (ii) [15] proposed the Relief filter approach and SVM for classifying levee passive seismic signals in the earth dam, (iii) [16] used filter (mutual information and statistical dependence) methods and embedded (cross-validation and pruning) methods to classify volcano-seismic signals with k-Nearest Neighbors and Decision-Trees, (iv) [17] employed Information Gain filter method with ANN to predict earthquakes. Although the aforementioned studies demonstrated that filter-based feature selection is successful at enhancing categorisation outcomes in a variety of seismic signals, filter methods are often unable to identify the discriminate features, such as those associated with long tail distribution. Additionally, the filter methods do not eliminate multicollinearity (a statistical concept where several independent variables in a model are correlated), which could result in the selected features being suboptimal for signal discrimination. Recently, wrapper methods were also employed to enhance volcano-seismic signals classification [18,19], based on the inferences from the classification model, and the performance surpasses the filter-based methods, while usually confined to a high level of computational complexity and subjects the model to overfitting.

Compared to supervised learning with aforementioned feature selection, semi-supervised learning only requires a small quantity of labeled event data, which could reduce the effects of human error due to labeling uncertainty, while obtaining relatively high accuracy [20]. Motivated by recent successes of graph-based semi-supervised learning, mainly focused on 2D and 3D image data [21,22], we focus on graph-based semi-supervised learning, due to its ability to handle classes with arbitrary signal distribution generating a smooth feature subspace. Graph learning refers to finding a signal representation via a graph, relying on either statistical methods or spectral graph methods, based on data observations to represent the signal in a low-dimensional subspace [23]. Graph spectral-based feature weight learning of [21] assigns a feature importance score to each feature assuming feature independence. In [21] constructed/extracted feature vectors are embedded onto a representation graph, where the distances between detected signals are assessed with the feature vectors and a critical parameter known as graph kernel bandwidth, which is usually manually set. Appropriate estimation of graph kernel bandwidth is essential for graph signal representation but challenging. Some recent studies on optimising graph kernel bandwidth, reported in [22,24,25], are either tied to a specific problem or might be affected by the randomness of feature pair selection. In [26], an iterative, alternating feature learning and classification approach was proposed for characterising slide quakes, earthquakes, tremors and calibration shots from a relatively less noisy dataset and considering only one channel.

This paper goes beyond [26] by proposing a comprehensive and integrated seismic monitoring workflow for the continuous multi-channel recorded signal at an ongoing landslide, which consists of multi-channel event detection with linear coherency analysis via MCM, graph-based feature weight optimisation and classification. Specifically, with the continuous recordings from multiple sensors in an array, the proposed system first detects potential events with the coherence analysis (i.e., MCM) of the multi-channels, and identifies the strongest signal components for feature construction. Then, we design graph-based feature weight optimisation for landslide-induced event classification, comprising, in addition to earthquakes, endogenous events such as rockfalls and seismic sources related to landslide processes (e.g., fissure formation) thereafter referred to as slide quakes, using the same terminology of [12]. These types of events tend to be localised, channel stations are relatively close for such studies and therefore time difference in signal arrival at different events is negligible for the purpose of multi-channel event detection and classification. Technically, we enhance the iterative, alternating feature weight optimisation and classification approach of [21] (see also [26]) with a new dual problem to reduce the computational complexity of the algorithm.

Briefly, the contributions of this paper can be summarised as follows:

1. To mitigate the impact of variable background noise from the multi-channel recordings, we propose a detection scheme that combines MCM coherence analysis and Neyman–Pearson lemma (see Section 2.1 and Algorithm 1), so that the seismic events recorded by a portion of the channels are readily identifiable, which is more in line with practical application scenarios as well;
2. We adapted the graph-based feature weight optimisation approach of [21] for seismic signal classification and proposed a new graph kernel bandwidth optimisation method to learn the best representation graph (Section 2.2);
3. We assess the proposed detection scheme and graph-based feature weight optimisation, respectively, on the cataloged event as in [12] and the continuous recordings (24–28/November/2014), manually labeled by a skilled expert; the results outperformed STA/LTA algorithm applied in the frequency domain, as in [12], showing the potential of the proposed detector (Section 3.2)
4. We explore in detail the impact of feature engineering (filter, wrapper, embedded-based feature selection approaches, feature extraction with PCA, and adapted graph-based feature weight optimisation) on landslide-induced signal classification (Section 2.2 and Algorithm 2), and conclude that with graph smoothness, the features highlighted with adapted graph-based feature weight optimisation are more discriminative (Section 3.3 and Section 3.4);
5. Finally, following graph-based feature weight optimisation, we contribute a feature recommendation list of rockfall, slide quake, earthquake, and natural and anthropogenic noise occurrences, which summarise the most distinct characteristics of each of the aforementioned signal classes (Section 4).

The rest of this paper is structured as follows. Section 2 discusses the proposed methodology, which includes multi-channel detection and graph-based feature weight optimisation and classification. In Section 3, an overview of the experimental design and the outcomes achieved with the seismic dataset recorded at the Super-Sauze active landslide site are given. Section 4 discusses feature recommendations for particular types of seismic signals together with the geological interpretation. Section 5 contains the conclusion and future works. Finally, the appendix provides a complete table of generated features.

## 2. Methodology

**Figure 1:**
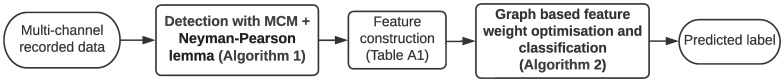
Workflow of the proposed system. The proposed Algorithms 1 and 2 are in bold.

In this section, we describe our methodology. The workflow of the proposed system is shown in Figure 1. In the following, we describe each block of the system, one by one.

### 2.1. Multi-Channel Detection

Seismic monitoring is regularly performed with multiple sensors deployed over the area of interest to continuously record the activities over vast distances of the order of kilometers. However, depending on the relative distance of the source to sensors, some sensors may not record a particular event at a sufficiently large SNR to be identifiable. Combining the readings from multiple sensors has been shown to improve event detection, e.g., by stacking signals from multiple channels as in [27].

For the detection stage, we only consider the multi-channel recorded signals $X\in R^{C\times N}$, where *C* and *N*, respectively, represent the number of vertical channels and signal samples in that channel. During preprocessing, we normalise and filter the recorded signal to minimise the effect of signal attenuation and measurement noise. As the first step of detection, we fragment the recorded data X into non-overlapped length-*l* windows $W\in \left\{W_{1},\ldots ,W_{I}\right\}$, $W_{i}\in R^{C\times l}$, where the total number of windows is $I=\frac{N}{l}$. Then, we analyse the linear coherence across each vertical channel $c\in \left\{1,\ldots ,C\right\}$ (i.e., traces from different deployed stations) within window $W_{i}$ using MCM to form stacked signal $r_{i}$.

Next, similar to [11], Neyman–Pearson lemma removes the stacked signals $r_{i}$ that most likely contain only background noise with low SNR. After concatenating the remaining consecutive windows, we form new windows ${\tilde{W}}_{j}$. For example, suppose that $W_{1}$, $W_{2}$, $W_{3}$, $W_{5}$, and $W_{6}$ are the remaining windows after Neyman–Pearson lemma. ${\tilde{W}}_{1}$ is formed by concatenating $W_{1}$, $W_{2}$, $W_{3}$ and ${\tilde{W}}_{2}$ is formed by concatenating $W_{5}$ and $W_{6}$. Let ${\tilde{w}}_{c}^{j}$ denote the channel *c*’s samples of ${\tilde{W}}_{j}$. In the final step, we automatically select the best channel segments ${\tilde{w}}_{c^{*}}^{j}$ (i.e., the ones that maximise SNR among all channels within each ${\tilde{W}}_{j}$) as the detected events to feed to the feature construction step (see Figure 1). The proposed multi-channel detection is summarised in Algorithm 1.
**Algorithm 1:** Multi-channel detection**Require:**  Recorded multi-channel data ***X***, Window length *l*, No. of channels *C***Ensure:** Detected events1:**Preprocessing:** Filter ***X*** with a (5–100 Hz) bandpass filter; and split it into non-overlapping windows ***W**_i_* of length *l*2:For each ***W**_i_*, use MCM [27] to obtain *r_i_*3:Set threshold Γ ← using Equation (10) of [11]4:Keep windows ***W**_i_* for which *r_i_* > Γ, and concatenate all such consecutive windows, to form new window ${\tilde{W}}_{j}$ with ${\tilde{w}}_{c}^{j}$ denoting its *c*-channel5:**for** each window ${\tilde{W}}_{j}$ **do**6: Identify channel c^*^ that maximises SNR across all ${\tilde{w}}_{c}^{j}$, *c*∈{1, …, *C*} [11]7: **return** The detected events ${\tilde{w}}_{c^{*}}^{j}$.

The choice of time window length *l* should consider the trade-off between noise suppression and time resolution [27]. In our case, the duration of the target signals vary from 0.5–100 s, while the majority are within the range 1–2 s, thus l=0.2s is chosen as length of all windows $W_{i}$. MCM [27] is then used on $W_{i}$ to obtain a stacked signal $r_{i}$ (Step 2), where we set the number of channels for signal linear coherency analysis to 3, as in [27]. If the stacked signal amplitude $r_{i}$ exceeds the calculated threshold Γ (Step 3) with Neyman–Pearson lemma, the windows are kept and concatenated to form ${\tilde{W}}_{j}$ (Step 4). Finally, for each ${\tilde{W}}_{j}$, the detected segments ${\tilde{w}}_{c^{*}}^{j}$ are obtained from a channel $c^{*}$ that maximises the SNR over all *C* channels [11].

### 2.2. Graph-Based Feature Weight Optimisation and Classification

After events are detected from multi-channel recorded data, as discussed in previous subsection, we construct K=119 features as shown in Table A1, in Appendix A, for each detected event ${\tilde{w}}_{c^{*}}^{j}$. The signal temporal, spectral, cepstrum, and acoustic features are calculated, and the polarity attributes are calculated independently on the three-component seismometers. Then we embed the constructed features into a connected, “representation” graph, G=(V,A), where V is the set of vertices and A is the graph adjacency matrix [11]. Each vertex in the graph ν∈V corresponds to one of the detected events and is characterised by the corresponding *K*-dimensional feature vector. The graph needs to represent well the relationships between the events, and is learnt based on the importance of the features, as described next.

Let $f_{k}\left(i\right)$ represent the *k*-th feature of event *i* assigned to vertex $\nu_{i}$. Then, we set the (i,j) entry in A, $a_{i,j}$, i.e., the weight of the edge between nodes *i* and *j*, as:

(1) $$a_{i,j}=exp\left\{-\overset{K}{\underset{k=1}{\sum }}\frac{F_{k}\left(i,j\right)}{2\sigma_{k}^{2}}\right\},$$

where $\sigma_{k}$ represents the graph kernel bandwidth for the *k*-th feature (i.e., $\frac{1}{2{\sigma_{k}}^{2}}$ is the weight of feature *k*), and $F_{k}\left(i,j\right)={\left(f_{k}\left(i\right)-f_{k}\left(j\right)\right)}^{2}$ is the squared Euclidean distance of feature *k* between events (nodes) *i* and *j*.

We assign to each node, a discrete graph signal s that carries the class label of the corresponding event in the training set and zero for nodes corresponding to test set, as below:

(2) $$s_{i}=\left\{\begin{array}{cc} +1, & \mathrm{if}\;\mathrm{Event}\;i\;\mathrm{belongs}\;\mathrm{to}\;\mathrm{the}\;\mathrm{target}\;\mathrm{Class}\;\mathrm{and}\;i\leq n\\ -1, & \mathrm{otherwise},\;\mathrm{and}\;i\leq n\\ 0, & \mathrm{for}\;n<i\leq N \end{array}\right.,$$

where *n* is the number of events in the training dataset. Please note that we are using multiple binary classifiers—one for each class. Therefore above, $s_{i}$ corresponding to the training set can take only two values +1 (indicating class membership) and −1 (indicating not class membership).

This way, if graph G captures well correlation between the events, then the nodes with the same label will be connected by high-weight edges, i.e., the *N*-length graph signal s will be piecewise smooth with respect to G and we can extrapolate the missing labels (that are initialised to zero), e.g., via GLR, by finding the smoothest graph signal that fits the training data [26].

**Figure 2:**
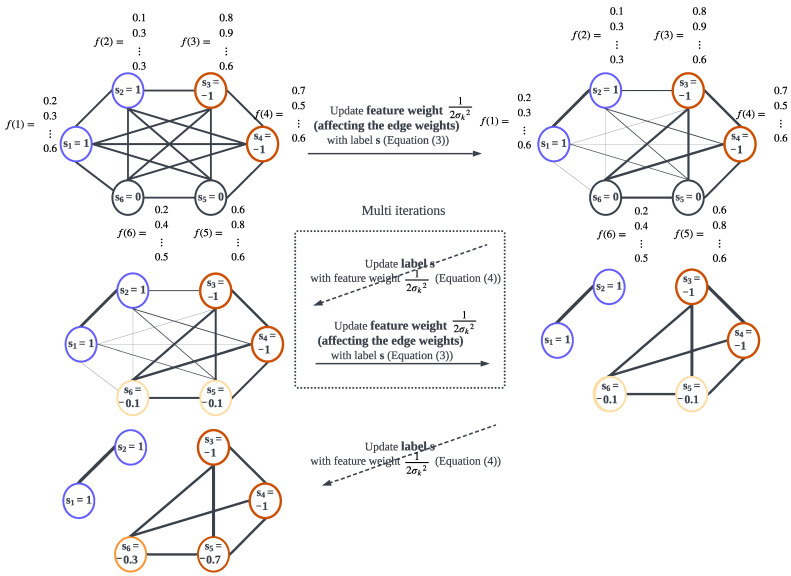
Graph-based feature weight optimisation and classification (schematic diagram), where the solid line represents Graph-based feature weight optimisation, and dashed line represents Graph-based classification.

We provide an illustration (a toy example) in Figure 2 to further clarify the graph-based feature weight optimisation and classification approach, where we build a fully connected graph with six nodes that correspond to the detected events. Specifically, in Figure 2, four nodes are used for training, i.e., $s_{1},s_{2}=1$, $s_{3},s_{4}=-1$, denoting that the first two nodes correspond to Class 1 (blue circles) and the third and fourth nodes do not belong to Class 1 (red circles) events while nodes five and six (yellow) are used for testing and are initialised to $s_{5},s_{6}=0$. As illustrated in Figure 2, the whole workflow consists of two steps: (1) Graph-based feature weight optimisation (shown as a solid line in Figure 2) based on feature vector set $F\in \left\{f(1),\ldots ,f(N)\right\}$, where $f\left(i\right)=[f_{1}\left(i\right),\ldots ,f_{K}\left(i\right)]$; here we consider the optimisation problem as in Equation (3) to determine the feature weight $\frac{1}{2{\sigma_{k}}^{2}}$ and then the graph edge weights are obtained with Equation (1) (a thicker edge indicates that the connected events have higher correlation), (2) graph-based classification, (dashed line in Figure 2), with the optimised edge weights; the labels of the testing events $s_{5}$ and $s_{6}$ are obtained with Equation (4). The above two steps alternatively and iteratively update the feature weights and event labels until the stopping criteria. These two steps are described next in more detail.

#### 2.2.1. Graph-Based Feature Weight Optimisation

In this subsection, we provide a detailed description of the adapted graph feature weight optimisation (the solid line step in Figure 2), which was first proposed in [21]. Specifically, the algorithm, shown as Algorithm 2, represents the iterative alternating binary classification via (Normalised) Graph Laplacian Regularisation classifier ((norm)GLR) [26] or with Graph Total Variation (GTV) [23], and graph-based feature weight optimisation via:

(3) $$\arg\min_{\sigma_{k}}\underset{i,j}{\sum }exp\left\{-\overset{K}{\underset{k=1}{\sum }}\frac{F_{k}\left(i,j\right)}{2\sigma_{k}^{2}}\right\}e_{i,j}+U\left(\sigma \right),$$

where $e_{i,j}={\left({\tilde{s}}_{i}-{\tilde{s}}_{j}\right)}^{2}$, ${\tilde{s}}_{i}$, ${\tilde{s}}_{j}$ are the predicted graph signal, and U(σ) is an indicator function that returns 0 if all elements of $\sigma =[\sigma_{1}\ldots \sigma_{K}]$ are in the range (0,1], or ∞, otherwise. The algorithm is initialised to $\sigma_{k}=0.7$ (Step 2) and then adapts the graph, i.e., $\sigma_{k}$, via feature selection and prioritisation by minimising Equation (3) using gradient descent (Steps 7 and 10). The optimisation problem Equation (3) subjects to $\sum_{k}\sigma_{k}\in \left(0,\gamma \right]$.
**Algorithm 2:** Alternative Graph-based Feature Weight Optimisation and Classification**Require:**  Constructed feature set F  Initial graph signal ***s***, tolerance *μ*, *ϵ*, constant *γ*, step size *θ***Ensure:** The predicted label $\tilde{s}$, feature weight $\frac{1}{2{\tilde{\sigma }}^{2}}$1:**Initialization**: *t* = *ϵ*;2:Initialise Graph kernel bandwidth ***σ**_k_*3:**while***t* > *μ* **do**4:  ***A*** ← Equation (1) by feeding F; update H5:  ${\tilde{s}}_{n+1:N}\leftarrow$ Equation (6); $\hat{t}=\epsilon$6:  $m={\tilde{s}}^{⊤}H\tilde{s}$7:  ∇ ← via gradient descent Equation (3) [21]; *j* = 08:  **while** $\hat{t}>\mu$ **do**9:    **if** *j* > 0 **then**10:     ∇ ← via gradient descent Equation (3) [21]11:    $\forall k,\tilde{\sigma_{k}}=\sigma_{k}-\theta \times \nabla$; j=j+112:    $q={\tilde{s}}^{⊤}H\tilde{s}$13:    update ***A*** ← Equation (1) using F and $\tilde{\sigma }$; update H14:    $\hat{q}={\tilde{s}}^{⊤}H\tilde{s}$15:    $\hat{t}=\hat{q}-q$16:  ${\tilde{s}}_{n+1:N}\leftarrow$ Equation (6) with updated H17:  $\hat{m}={\tilde{s}}^{⊤}H\tilde{s}$18:  $t=\hat{m}-m$19:**return** The predicted label $\tilde{s}$ and feature weight $\frac{1}{2{\tilde{\sigma }}^{2}}$

Feature selection is performed iteratively between feature weight update (Step 11) and classification (Steps 12 and 14). The definition of H in Algorithm 2 (Step 4) depends on the classifier used. Specifically, H represents the combinatorial graph Laplacian matrix for GLR classifier defined as L=D−A (D is the diagonal matrix, given by $d_{i}{,}_{i}=\Sigma_{j}a_{i}{,}_{j}$); its normalised form, $L_{norm}=D^{\left(-1/2\right)}LD^{\left(-1/2\right)}$ for the normGLR classifier, or for the GTV classifier $\tilde{A}={\left(I-A\right)}^{H}\left(I-A\right)$ (I is the identity matrix, and ^*H*^ represents the Hermitian operator). The hyperparameter γ=119, the tolerance μ=0.0001, and ϵ=10,000 are heuristically set; additionaly, the step size θ set as the one over the Lipschitz constant. Please note that $\sigma_{k}\geq 0$ represents the feature weight given to feature *k*, where smaller $\sigma_{k}$ indicates that *k*-th feature is more important. Thus, the algorithm inherently performs feature selection.

#### 2.2.2. Graph-Based Classification

The graph classification problem, i.e., finding the smoothest graph that spans training and testing nodes and fits the training labels, can be solved via quadratic formulation as in [11], which is fast but with poor worst-case errors and requires finding a pseudo-inverse of potentially large Laplacian matrix. Alternatively, NP-hard quadratic constrained quadratic relaxation and semi-definition relaxation (SDR) are known to provide good error-bounded approximations [21]. Therefore, we use graph-based classifiers with SDR, and following the idea of [21], the three graph-based classifiers can be solved by SDR with a general formation:

(4) $$\begin{array}{cc} \min_{S,s} & tr(HS),\\ s.t. & S_{i,i}=1,\;i\in \left\{1,\ldots ,N\right\},\\ & \left[\begin{array}{cc} S & s\\ s^{⊤} & 1 \end{array}\right]⪰0,\;s_{i}={\hat{s}}_{i},\;i\in \left\{1,\ldots ,n\right\}, \end{array}$$

where tr(·) is the trace operator, ${\hat{s}}_{i}$ denotes the label of the *i*-th event in the training set, with n<N denoting the size of the training set.

Optimisation problem (4) is of polynomial complexity, thus we convert (4) to its dual problem [21], defined as:

(5) $$\begin{array}{cc} \lambda^{*}= & \max_{\lambda }-1^{⊤}\lambda \\ & s.t.\;H+diag(\lambda )⪰0, \end{array}$$

where λ is the Lagrange multiplier. With the obtained optimised $\lambda^{*}$, the optimised solution of (4) is equivalent to finding the infimum of Lagrange function $La(s,\lambda^{*})$:

(6) $$\begin{array}{cc} La & \left(s,\lambda^{*}\right)\\ = & s^{⊤}\left(H+diag\left(\lambda^{*}\right)\right)s-\lambda^{*}1\\ = & s^{⊤}H^{*}s-\lambda^{*}1\\ = & \left[\begin{array}{cc} s_{1} & s_{2} \end{array}\right]\left[\begin{array}{cc} {H_{11}}^{*} & {H_{12}}^{*}\\ {H_{21}}^{*} & {H_{22}}^{*} \end{array}\right]\left[\begin{array}{c} s_{1}\\ s_{2} \end{array}\right]-\lambda^{*}1\\ = & s_{1}^{⊤}{H_{11}}^{*}s_{1}+\left(b^{⊤}+c\right)s_{2}+s_{2}^{⊤}{H_{22}}^{*}s_{2}-\lambda^{*}1, \end{array}$$

where $s_{1}=s_{1:n}$, $s_{2}=s_{n+1:N}$, $H^{*}=H+diag\left(\lambda^{*}\right)$, $b={{H_{12}}^{*}}^{⊤}s_{1}$ and $c={s_{1}}^{⊤}{H_{12}}^{*}$. By setting the first-order derivative of the Lagrange function La to zeros, we obtain the solution ${s_{2}}^{*}=\frac{-{H_{22}}^{-1}\left(b+c^{⊤}\right)}{2}$.

## 3. Experimental Setup and Results

### 3.1. Dataset

We used the publicly available raw seismic recordings accessed on 8 January 2022 (https://seismology.resif.fr/networks/#/MT), sampled at Fs=250Hz, in the periods 11 October–19 November 2013, 10–30 November 2014, and 9 June–15 August 2015, from the permanent tripartite array SZC, with 40m layout, of the French Landslide Observatory OMIV (Observatoire Multi-disciplinaire des Instabilités de Versants). Since there were no missing data during the aforementioned period, the recordings on array SZC met the criteria for analysis of continuously recorded data in this study. The array comprises one 3-component center site and three vertical 1-component sites organised as an equilateral triangle. Algorithm 1 takes as input the continuous signals from the 4 vertical channels. A catalog of manually verified events, detected with spectrogram analysis, from [12], for this period, was used for labeling. We used the same labels for the 4 classes in the catalog, and removed duplicate events. This results in 401 “Rockfall”, 234 “Slide Quake”, 388 “Earthquake”, and 351 “Natural/Anthropogenic noise”, totalling 1374 events, which are all considered in this paper.

### 3.2. Multi-Channel Detection

**Figure 3:**
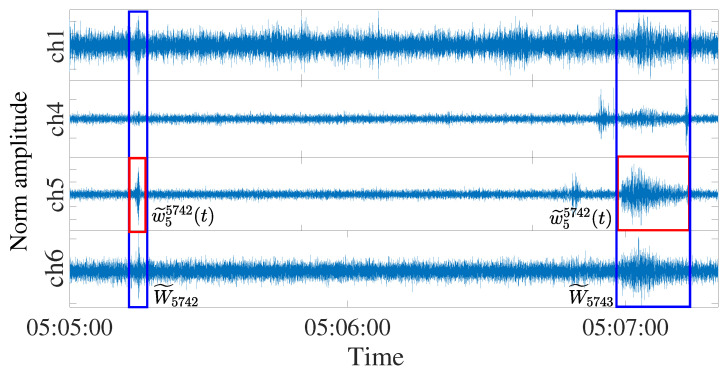
Example of the kept ${\tilde{W}}_{5742\&5743}$ (blue rectangular box) and ${\tilde{w}}_{5}^{5742\&5743}$ (red rectangular box) as given in Algorithm 1 (Period: 03/November/2013 (05:05:00–05:07:30)).

Raw recorded data from the vertical channels are fed into Algorithm 1 that selects the optimum channel segments that are least affected by noise and removes the segments with low signal activity, where noise is predominant. An example of Algorithm 1 output is shown in Figure 3 for four bandpass filtered vertical channels for the same window. With threshold $\Gamma =800\;{\mathrm{nm}}^{3}/s^{3}$, two stacks at times 05:05:13 and 05:06:59 are deemed of interest. These are highlighted in blue rectangular box (kept windows) in Figure 3, where the amplitude of each channel is scaled to [−1,1]. Step 6 identified the optimal channel (the one with highest SNR) $c^{*}$ is ch5 for both windows ${\tilde{W}}_{5742}$ and ${\tilde{W}}_{5743}$, thus, red segments ${\tilde{w}}_{5}^{5742}$ and ${\tilde{w}}_{5}^{5743}$ are selected.

#### 3.2.1. Detection on Cataloged Events

To demonstrate the effectiveness of Algorithm 1, we benchmark its performance against the single channel detection approach of [11] using cataloged events [12]. As shown in Table 1, compared to [11], Algorithm 1 detected more cataloged events (shown as True positives), missed fewer cataloged events (shown as false negatives) and detected fewer additional events (shown as false positives) that were not present in the catalog. The 23 events missed by Algorithm 1 comprised 2 rockfalls, 3 slide quakes, 1 earthquake, and 17 noise events. The 28,555 detected events that were not cataloged are not necessarily non-endogenous or non-seismic events, and could have been missed during manual labeling at cataloging stage. Therefore, Algorithm 1 is effective in maximising detection of cataloged events, missing fewer events, and minimising detection of uncataloged events.

#### 3.2.2. Detection on Continuous Data Recorded between 24 and 28/November/2014

To further validate the performance of the proposed multi-channel detection, we asked an expert to manually identify the seismic events on multi-channel continuous recorded data between 24/November/2014 and 28/November/2014. The period of time was chosen because, according to the catalog of [12], all four events are present in this time period (65 rockfalls, 18 slide quakes, 23 earthquakes and 14 noise), which were detected with an STA/LTA algorithm applied in the frequency domain.

As illustrated in Table 2, 1006 events are newly identified by the expert, which was missed with STA/LTA algorithm applied in the frequency domain [12]. A total of 614 of these 1006 events have been detected using proposed Algorithm 1, and these include 6 rockfalls, 3 slide quakes, 2 earthquakes and 603 noise events that were missed by STA/LTA in [12]. This further demonstrates the effectiveness of our detection technique. Additionally, the majority of the 392 (392 = 1006 − 614) missed occurrences by our proposed multi-channel detection were 295 noise events, and only 45 rockfalls, 48 slide quakes and 4 earthquakes. The difficulty of recognising rockfall and earthquake events arises since they are regarded as endogenous landslide seismicities with varying SNR. The limitations of the proposed detection approach and future work will be discussed later.

### 3.3. Feature Engineering

In this section, we compare the output of conventional feature extraction and selection, as discussed in Section 1, to that of the adapted graph feature weight optimisation, described in Section 2.2. For each detected event segment from Algorithm 1, K=119 features are constructed, as listed in Table A1 in the Appendix A. During feature extraction via PCA, we select the top 95% of the principal components, amounting to, on average, 44 principal components per class, which are then used by the benchmarked SVM and RF classifiers.

We evaluate the filter, wrapper, and embedded feature selection methods from feature selection library FsLib accessed on 8 January 2022 (https://www.mathworks.com/matlabcentral/fileexchange/56937-feature-selection-library) [28]. For each class of signals, a sorted feature vector (high to low rank) is generated by each FsLib feature selection approach. The optimal feature space subset *o* and |o|∈[1,…,K] is selected using 10-fold cross-validation at the classification training stage. The optimal *o*, for each of five used classifiers (RF, SVM, GLR, normGLR and GTV), is the one that results in the highest validation accuracy for each classifier. If the highest accuracy score corresponds to several different feature subsets, then the optimal subset is chosen as the one with the minimum dimension. The feature selection result via FsLib shows the optimal feature selection approaches, which are embedded fsv (|o|=45 for rockfall, and |o|=21 for earthquake, i.e., 45 and 21 features are selected for these two classes, respectively), filter mutinfffs (|o|=38 for slide quake, and |o|=53 for noise) and *o* is common to all 5 classifiers. Similarly, for the graph-based feature weight optimisation (GLR, normGLR and GTV), we adapt 10-fold cross-validation with training data to tune the parameters in Algorithm 2, and the optimal feature weight $\frac{1}{{\tilde{2\sigma }}_{k}^{2}}$ is the one which results in the best classification accuracy. Since the optimal feature weight is observed to be similar for all three classifiers, we use the average over 10 folds from all three classifiers.

**Figure 4:**
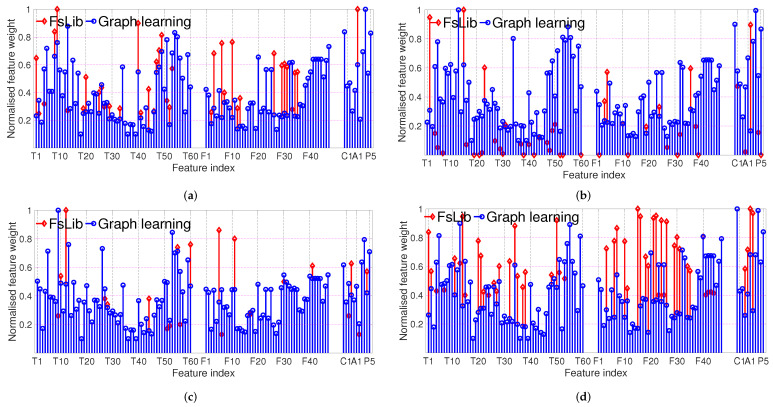
Normalised feature weights from the resulting set *o* for all 4 classes. Features of high importance include temporal (T), Spectral (F), Cepstrum (C), Acoustic (A), Polarity (P). (**a**) Feature weight for rockfall. (**b**) Feature weight for slide quake. (**c**) Feature weight for earthquake. (**d**) Feature weight for noise.

The resulting set *o* for both FsLib feature selection and graph-based feature weight optimisation for each class is shown in Figure 4, in red and blue, respectively. The feature weights are normalised [0,1]. We observe a good mix of temporal, spectral and cepstral features selected for all classes. FsLib tends to discard the less discriminative features, unlike graph-based feature weight optimisation which does not discard features but tends to give a much higher weight to the discriminative features, with the highest feature weight at 1 and the least discriminative feature weight around 0.1. The selected feature set *o* for FsLib and graph-based feature weight optimisation are very similar.

**Figure 5:**
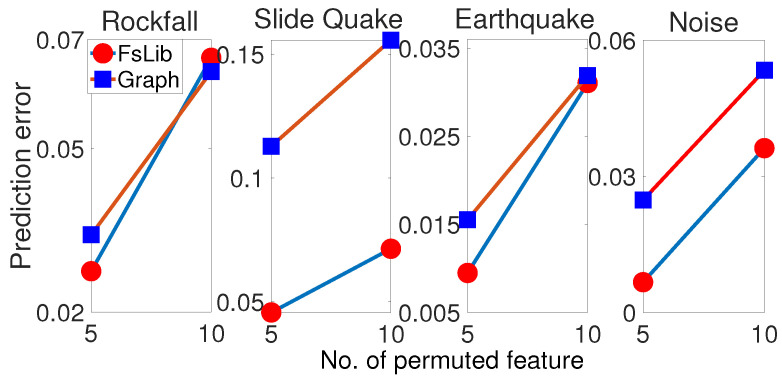
Permutation Feature Importance. The horizontal axis denotes the feature number starting from the most important feature for each of the four classes. The vertical axis is the decrease in sensitivity measure $\Delta se_{h}$.

Next, the feature selection performance is evaluated via the permutation feature importance method, by looking at how fast the prediction error increases after permuting the features. The larger the gradient, the higher the importance of the permuted features. Figure 5 shows the results with the normGLR classifier. We consider random permutation of 5 and 10 most important features (as per Figure 4) that correspond to numbers 5 and 10, in the horizontal axis. Specifically, (1) with the highlighted optimal subset of features *o* both FsLib and graph-based feature weight optimisation, the top *h* (h=5 or 10) features are permuted randomly forming a new feature set; (2) then, classification is performed with the newly formed feature sets, resulting in sensitivity score $se_{h}$; finally, (3) the prediction error is calculated as $\Delta se_{h}=se_{0}-se_{h}$, where $se_{0}$ denotes the sensitive score without any permutation. The values $\Delta se_{h}$ are then plotted in Figure 5 against the number of permuted features *h*. Since the processing involves random permutations, $se_{h}$ is obtained after averaging the result of 10 runs.

As shown in Figure 5, permuting the selected features by both FsLib and graph-based feature weight optimisation results in an increase in $\Delta se_{h}$, especially for slide quake, which indicates the effectiveness of the feature selection process. Furthermore, comparing graph-based feature weight optimisation and FsLib, it can be concluded that the highly ranked features by graph-based feature weight optimisation are more discriminative because they generally correspond to higher prediction errors due to the removal of these highly ranked features.

### 3.4. Classification

We first evaluate the effect of aforementioned feature engineering approaches (extraction via PCA, selection via FsLib and graph-based feature weight optimisation) on classification performance for the five classifiers of interest, considering only cataloged events. Afterwards, we present the classification results for the proposed workflow, as illustrated in Figure 1 for continuous data.

#### 3.4.1. Effect of Feature Engineering on Classification Performance

Using only cataloged event segments, we adopt the one-against-all classification strategy with 10-fold cross-validation. For each class, we randomly split the training and testing set with a 70:30 ratio. The test is carried out 50 times under identical conditions to ensure repeatability of results, and the mean and standard deviation of the Sensitivity measure are shown in Table 3.

As in [12], we present classification performance with the sensitivity measure, equivalent to Recall, that is the ratio of correct events predicted over total number of cataloged events for each class. We present the confusion matrix to explain misclassification, as shown in Table 4 and Table 5 for the feature construction only and for feature construction with feature selection steps, respectively.

An RF classifier (tree bagger version similar to the one used in this paper) with feature construction and selection was used with the same cataloged dataset in [12]. Sensitivity results in [12] (rockfall 0.94, slide quake 0.93, earthquake 0.94, and noise 0.92) were provided for a balanced testing set comprising 70 events per class, while our dataset is unbalanced, comprising 30% of the cataloged classes, which is a more realistic scenario. The 71 constructed features used for classification included nine network geometry attributes, such as the station with the highest SNR, which we did not consider since it lacks generalisation. It is noted that the mean sensitivity over all classes falls to 0.9 without these network geometry attributes, ranging from 0.86 to 0.94. Our replicated RF results, as observed on the RF with FsLib selected features in Table 3, are in agreement.

As shown in Table 3 and Table 4, there is no performance benefit performing PCA for either RF or SVM classifier compared to using constructed features. The only benefit is lower complexity due to dimensionality reduction: 119 features vs around 44 principal components, fed to the classifier. Indeed, with only constructed features fed to the classifier, all classifiers have similar performance. However, although we do not observe performance improvement for RF or SVM with the additional FsLib feature selection step, we do observe significant performance improvement for graph-based classifiers. The benefit of feature selection for SVM and RF is dimensionality reduction by more than 50% of the feature set per class from 119 to |o|=45 for rockfall, 38 for slide quake, 21 for earthquake and 58 for noise. As expected, we also observe even better performance improvement for feature selection via the proposed graph-based feature weight optimisation for the graph-based classifiers. Thus, feature selection is considered a beneficial step for classification, both for performance improvement and complexity reduction. The performance improvement due to proposed Algorithm 2 can be explained via the confusion matrix shown in Table 5. Compared to Table 4, for rockfall, the more discriminate feature selection shows that rockfalls and slide quakes are not confused with earthquakes anymore.

The cataloged nature/anthropogenic noise events are miscellaneous, caused by human-made activities including footsteps to environmental conditions such as storms, with relatively not-very-distinct features compared to the other 3 classes, as shown in Figure 4. Thus, the noise class has the worst sensitivity, but even noise performance is improved with graph-based feature weight optimisation since noise signals are less likely to be confused with rockfalls. Earthquakes usually have high SNR, with distinct P and S wave arrivals, which make them less likely to be confused with any other class, resulting in around 0.96 sensitivity for all classifiers, with or without, feature selection. As stated in [12], and as observed in Table 5, it is sometimes difficult to distinguish slide quakes from small-volume rockfalls, rockfalls as footsteps (noise).

#### 3.4.2. Performance Comparison of the Graph-Based Classifiers against Benchmarks

The performance of graph-based classifiers (norm)GLR and GTV are highly dependent on the graph kernel bandwidth $\sigma_{k}$ in Equation (1). A too-small value of the bandwidth would lead to a poor representation of the local structures; conversely, a considerable high value could result in a coarse description of the data. As illustrated in Table 3, with constructed features, graph-based classifiers have similar performance to RF and SVM for rockfalls, slide quakes, and earthquakes, when graph kernel bandwidth is set as in [22]. However, with optimised feature weights obtained with benchmarked feature selection and graph-based feature weight optimisation, the potential of graph-based classifiers is maximised (the classification sensitivity increases by about 3% for rockfalls and slide quakes). For instance, the classification sensitivity for rockfalls with GLR classifiers reaches 92% (graph-based feature weight optimisation), 91% (benchmarked feature selection FsLib) compared to 89% (constructed feature only). In conclusion, graph-based classifiers outperform RF and SVM with appropriate graph kernel bandwidth (feature weight).

#### 3.4.3. Classification with Multi-Channel Detection (Algorithm 1) on Continuous Data Recorded between 24 and 28/November/2014

Here, we look at the more realistic scenario of classifying events from the continuous (vs. only cataloged) dataset which includes detection misses and uncataloged events as discussed in Section 3.2. We focus on the period 24–28/November/2014, which contained the most cataloged events, with 65 rockfalls, 18 slide quakes, 23 earthquakes and 14 noise. After detection, as per the proposed workflow of Figure 1, we missed only 1 noise event and detected 614 uncataloged events. With the 119 features constructed as shown in Table A1 for each event detected by Algorithm 1, the classification training set is formed as described in Section 3.4.1, without the cataloged event in the selected period. Specifically, we used the feature weight obtained by the graph-based feature weight optimisation to distinguish the cataloged events with normGLR classifier, resulting in classification performance indicated as a confusion matrix in Table 6. Performance is similar to that observed in Table 5.

As described in Section 3.2.2, after detecting signals from continuously recorded data, the result is manually evaluated by the expert. Here we assess the performance of the adapted graph-based feature weight optimisation and classification with expert verification over the selected period. The results are shown in Table 7, where we have another class which is considered not to belong to the four initial types of events. The adapted feature weight and classification workflow are resilient to rockfalls and slide quakes with only a minor reduction in classification sensitivity, according to Table 7. Additionally, it comes as no surprise that the classification results for earthquakes are unaffected; however, the situation is different for noise-type occurrences, which could be compensated with classification post-processing.

## 4. Feature Recommendation

Following the comprehensive classification performance evaluation, we observed that feature selection, rather than feature extraction via PCA, led to effective dimensionality reduction of the constructed feature set and performance improvement for different classes and classifiers. Here, we discuss which handcrafted optimised feature sets characterise slope failure endogenous events, including rockfalls, slide quakes, as well as earthquakes. In the following content, we list the common highlighted features observed by both recent research and our proposed graph-based feature weight optimisation in bold.

Using an RF classification of the same dataset on cataloged events, ref. [12] identified the following distinguishing attributes or features for our four classes of interest (without distinguishing unique features per class): Duration (T1), Ratio between ascending and descending time (**T8&9**), Energy in the first third part of the autocorrelation function (T38), Energy of the signal filtered in 50–100 Hz (T31), Mean and max of the DFT (**F1&2**), Central frequency of the 2nd quartile (**F4**), Energy in ([0, 1/4 ], [1/4, 1/2])*Fs (F12&13), Frequency at the max (F27), No. peaks in the curve showing the temporal evolution of the DFTs max, mean and median (F20&30, **F31**), Ratio between F20 and F31 (**F33**), No. peaks in the curve of the temporal evolution of the DFTs central frequency (**F34**) and Polarization azimuth (**P3**).

In their attempt to perform classification of slope failures, earthquakes, and noise on continuous data via an RF classifier, ref. [3] highlighted only the following eight most distinct features (without distinguishing unique features per class): Spectral gyration radius (F28), Spectral centroid (F8), Central frequency of the first quartile (F3), Variance of the normalised DFT (F10), Frequency at the maximum of the DFT (F27), Frequency at spectrum centroid (F29), Energy of the last two thirds of the autocorrelation function (T39) and Energy of the seismic signal in the frequency band of 1–3 Hz (**T28**).

Furthermore, for similar terrain and slope failures, through visual observation without automated feature selection and classification, ref. [29] characterised qualitatively the distinguishing features for each of our four classes of interest. Next, we list the distinguishing attributes identified by [29] and attempt to map them into the equivalent notation used in Table A1. *Rockfalls*: the falling block impacts produce spikes or jolts in the waveforms, which are visible both in the signal waveform as cigar shapes (final impact) (**T15**, T18, **T33**, and **T34**) and in the power spectral density function for most of the events (**T17**). *Slide Quakes*: short-duration (T1) (last less than 5 s) earthquake-like signals, with clearly discernable, trackable wave packets, emergent first arrivals, and undistinguishable P and S waves (**T2**, **T15**, **T22**, T33–36, and **T37**). *Earthquakes*: well-studied and potential landslide triggers that produce medium to long-duration signals (**T1**, **T9&10**, T38&39, and T41&42) with distinct P and S wave high impact arrivals. *Natural/Anthropogenic noise*: high-frequency range (>50 Hz) (**F12**, F13&14, **F16**) characteristics due to shallow installation of seismometers in clayey materials; duration, phase, and velocities of noise signals are not identifiable; furthermore, the noise waveform amplitude attenuation patterns are incoherent.

Although the discriminative features identified for landslide-induced events and earthquakes in [3,12,29] are not the same, they all show that temporal and spectral attributes are the most important for RF classifiers. More importantly, the aforementioned features that we also observe via our learning algorithms are highlighted in bold above. Additionally, we observed more discriminative features that improve the classification performance of RF, SVM and graph-based classifiers, highlighted in Figure 4 that we list next, per class.

### Feature Weight Analysis for Target Signals

*Rockfall*: the highly ranked features observed via FsLib and graph-based feature weight optimisation are: cross correlation-based features (T19–20, T25–27, T40, T47–51, T53–60, F39–48 (graph-based feature weight optimisation)), Energy of the signal filtered in 10–50 Hz (T29), dominant frequency (F7 FsLib), duration (T1 FsLib) No. of peaks in the curve showing the temporal evolution of the DFTs mean/median (F30&31 FsLib), waveform amplitude attenuation patterns (T4–7, T12&13 (graph-based feature weight optimisation)). The waveforms of rockfalls are variable due to the loose material saltation and flow combined with the moving character of the source.

*Slide quakes*: FsLib highlights the Optimum point of separation (T4), the max value of cross correlation with template earthquake (T16), the ratio between mean and median envelope signal (T23), and the max value of enveloped power density function (F5); while graph-based feature weight optimisation provided a high rank for the max value of cross correlation with template slide quake, rockfall, earthquake, (T12&13, T16), spectral features max envelope power density function (F5) and No. of peaks of the autocorrelation ac(f(v)) (F11).

*Earthquakes*: here, both FsLib and graph-based feature weight optimisation approaches provide more focused additional importance features: Dominant frequency (F7), cross correlation-based features (T53–60, (graph-based feature weight optimisation)), No. of peaks in the curve of the temporal evolution of the DFTs central and maximum frequency (F34&35 graph-based feature weight optimisation), cross correlation-based features (F39–48 (graph-based feature weight optimisation)) to capture the typical triangular-shaped sonogram pattern for earthquakes.

*Natural/Anthropogenic noise:* due to variability of the source of noise events |o|=53 for FsLib, with abundant complex spectral features such as F17–44, while graph-based feature weight optimisation results in highly ranking temporal features T1–10 and cross correlation-based features (T47–60).

Additionally, the following features are highly ranked for all four classes: Cepstral features C1 (standard deviation) and C3 (kurtosis); Acoustic feature A1 (No. of peaks linear prediction filter coefficient); Polarity features P2–5 (incidence angle, polarization azimuth, degree of linear polarization, degree of plane polarization).

**Figure 6:**
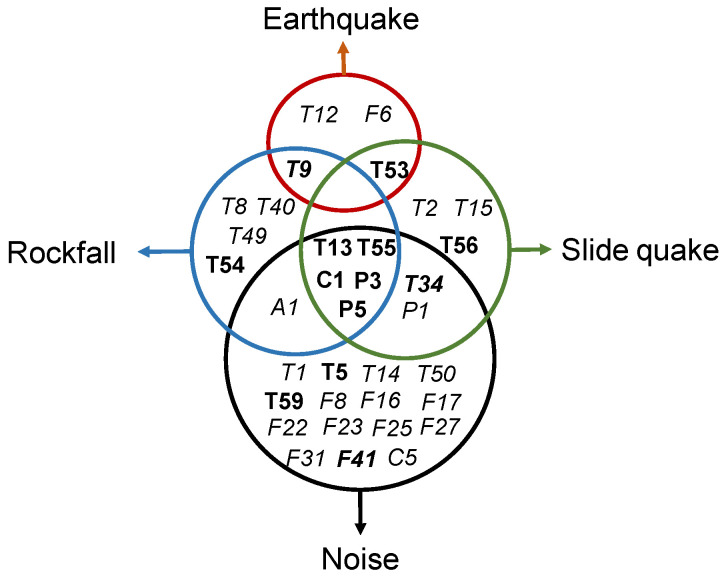
Features that best characterise rockfall, slide quake, earthquake, and environmental/anthropogenic noise. Graph-based feature weight optimisation features in bold, FsLib features in italics, and common features learnt by both graph-based feature weight optimisation and FsLib in bold italics.

We summarise the most distinguishing features, defined as those with normalised feature weight >0.8 (from Figure 4), in Figure 6.

## 5. Conclusions

In this paper, we address the challenges in automatically and accurately detecting and classifying slope failure endogenous events rockfall and slide quake (seismic sources related to landslide processes) as well as external sourced earthquake and Natural/Anthropogenic noise, through a novel automated workflow, which analyses large amounts of seismometer recordings from multiple channels/sensors. First, the proposed detection scheme combines MCM coherence analysis and Neyman–Pearson lemma to identify potential events and the representative signal segments from multiple channels to tackle continuous data containing interfering signals and low SNR slide quake events, without missing many cataloged events and detecting fewer uncatalogued events than processing a single channel would. Second, we evaluate graph-based feature weight optimisation and classification with a novel graph kernel bandwidth optimisation technique for characterising all four events, which are robust to lack of sufficient balanced data for training. Finally, after comprehensive experiments to demonstrate the impact of feature engineering including multi-feature selection and extraction approaches on classifiers depending on handcrafted features, we provide a detailed list of key features to consider for each of the four types of events. Additionally, we evaluate the proposed workflow on the raw continuous data recorded at the selected period (24–28/November/2014), manually labeled by an expert, where detection and classification with graph-based feature weight optimisation obtained very good results.

However, there are certain limitations in the proposed system that could be addressed in future work. First, the effectiveness of the used thresholding strategy depends on the consistency of the background noise distribution, hence certain low amplitude events are nevertheless missed by our detection scheme. Furthermore, due to their complicated and changing generating mechanism, the Natural/Anthropogenic noise categorisation findings need to be enhanced. Future research will therefore focus on adaptive background noise removal to increase SNR, identifying concealed microseismic activity, and using post-processing to enhance the classification outcome, particularly for natural/anthropogenic noise.

## Figures and Tables

**Table 1:** Detection on cataloged events for entire period.

	True Positives	False Negatives	False Positives
Detection [11]	978	396	108,288
Proposed detection (Algorithm 1)	1351	23	28,555

**Table 2:** Continuous detection result verification for the selected period.

	Catalog	Not Cataloged
Detection [12]	120	0
Manual expert detection	120	1006
Proposed detection (Algorithm 1)	119	614

**Table 3:** Sensitivity (mean and standard deviation).

Classifier	Rockfall	Slide Quake	Earthquake	Noise
RF (PCA)	0.84 (0.04)	0.76 (0.05)	0.95 (0.02)	0.80 (0.03)
SVM (PCA)	0.85 (0.04)	0.86 (0.04)	0.96 (0.02)	0.80 (0.03)
Constructed feature set (see Table A1)				
RF	0.89 (0.03)	0.84 (0.04)	0.96 (0.02)	**0.83 (0.03)**
SVM	0.87 (0.03)	0.86 (0.04)	0.96 (0.03)	0.82 (0.03)
GLR	0.89 (0.02)	0.85 (0.03)	0.96 (0.01)	0.70 (0.03)
normGLR	0.88 (0.02)	0.84 (0.02)	0.96 (0.01)	0.75 (0.02)
GTV	0.89 (0.01)	0.85 (0.03)	0.96 (0.01)	0.69 (0.03)
Feature selection via FsLib				
classifier	Rockfall	Slide Quake	Earthquake	Noise
RF	0.88 (0.03)	0.84 (0.04)	0.96 (0.02)	**0.83 (0.03)**
SVM	0.89 (0.03)	0.86 (0.04)	0.96 (0.02)	0.80 (0.04)
GLR	0.91 (0.01)	0.85 (0.03)	**0.97 (0.01)**	0.72 (0.02)
normGLR	**0.92 (0.02)**	**0.88 (0.02)**	**0.97 (0.01)**	0.79 (0.02)
GTV	0.84 (0.05)	0.84 (0.04)	0.96 (0.01)	0.75 (0.02)
Feature selection via graph-based feature weight optimisation (see Algorithm 2)				
classifier	Rockfall	Slide Quake	Earthquake	Noise
GLR	**0.92 (0.01)**	**0.88 (0.02)**	0.96 (0.01)	0.76 (0.02)
normGLR	0.91 (0.01)	**0.88 (0.02)**	**0.97 (0.01)**	0.80 (0.02)
GTV	**0.92 (0.01)**	0.87 (0.02)	0.96 (0.01)	0.75 (0.02)

**Table 4:** Mean Confusion Matrix (sensitivity score) for 50 runs (feature construction only) ^*a*^.

Classifier		Pred.Rockfall	Pred.Slide Quake	Pred.EQ	Pred.Noise
RF (PCA)	Ref.Rockfall	**0.84**	0.02	0.01	0.13
	Ref.Slide Quake	0.11	**0.76**	0.00	0.13
	Ref.EQ	0.02	0.01	**0.95**	0.02
	Ref.Noise	0.11	0.07	0.02	**0.80**
SVM (PCA)	Ref.Rockfall	**0.85**	0.02	0.00	0.13
	Ref.Slide Quake	0.05	**0.86**	0.01	0.08
	Ref.EQ	0.02	0.01	**0.96**	0.02
	Ref.Noise	0.10	0.08	0.02	**0.80**
RF	Ref.Rockfall	**0.89**	0.01	0.01	0.09
	Ref.Slide Quake	0.06	**0.84**	0.00	0.09
	Ref.EQ	0.02	0.02	**0.96**	0.01
	Ref.Noise	0.10	0.07	0.01	**0.83**
SVM	Ref.Rockfall	**0.87**	0.02	0.00	0.11
	Ref.Slide Quake	0.05	**0.86**	0.00	0.09
	Ref.EQ	0.01	0.02	**0.96**	0.01
	Ref.Noise	0.09	0.07	0.01	**0.82**
GLR	Ref.Rockfall	**0.89**	0.02	0.01	0.08
	Ref.Slide Quake	0.06	**0.85**	0.02	0.07
	Ref.EQ	0.02	0.01	**0.96**	0.02
	Ref.Noise	0.17	0.11	0.03	**0.70**
normGLR	Ref.Rockfall	**0.88**	0.02	0.01	0.09
	Ref.Slide Quake	0.06	**0.84**	0.01	0.09
	Ref.EQ	0.01	0.05	**0.96**	0.02
	Ref.Noise	0.15	0.09	0.02	**0.75**
GTV	Ref.Rockfall	**0.89**	0.02	0.02	0.07
	Ref.Slide Quake	0.06	**0.85**	0.03	0.07
	Ref.EQ	0.02	0.01	**0.96**	0.02
	Ref.Noise	0.15	0.10	0.05	**0.69**

^*a*^ The predicted (Pred.) events are represented with respect to the events of the reference cataloged (Ref.), EQ stands for earthquake.

**Table 5:** Mean Confusion Matrix (sensitivity score) averaged over 50 runs (FsLib and graph-based feature weight optimisation) ^*a*^.

Feature Selection via FsLib					
**Classifier**		**Pred.Rockfall**	**Pred.Slide Quake**	**Pred.EQ**	**Pred.Noise**
RF	Ref.Rockfall	**0.88**	0.01	0.01	0.09
	Ref.Slide Quake	0.07	**0.84**	0.00	0.09
	Ref.EQ	0.02	0.01	**0.96**	0.01
	Ref.Noise	0.09	0.08	0.00	**0.83**
SVM	Ref.Rockfall	**0.89**	0.01	0.01	0.09
	Ref.Slide Quake	0.04	**0.86**	0.01	0.09
	Ref.EQ	0.01	0.01	**0.96**	0.01
	Ref.Noise	0.10	0.08	0.01	**0.80**
GLR	Ref.Rockfall	**0.91**	0.02	0.01	0.07
	Ref.Slide Quake	0.05	**0.85**	0.01	0.10
	Ref.EQ	0.01	0.01	**0.97**	0.01
	Ref.Noise	0.14	0.13	0.01	**0.72**
normGLR	Ref.Rockfall	**0.92**	0.01	0.00	0.06
	Ref.Slide Quake	0.04	**0.88**	0.00	0.07
	Ref.EQ	0.01	0.01	**0.97**	0.01
	Ref.Noise	0.11	0.10	0.01	**0.79**
GTV	Ref.Rockfall	**0.84**	0.02	0.06	0.08
	Ref.Slide Quake	0.05	**0.84**	0.04	0.07
	Ref.EQ	0.01	0.02	**0.96**	0.01
	Ref.Noise	0.11	0.10	0.05	**0.75**
**Feature Selection via Graph-Based Feature Weight Optimisation**					
**Classifier**		**Pred.Rockfall**	**Pred.Slide Quake**	**Pred.EQ**	**Pred.Noise**
GLR	Ref.Rockfall	**0.92**	0.01	0.00	0.06
	Ref.Slide Quake	0.07	**0.88**	0.00	0.05
	Ref.EQ	0.03	0.01	**0.96**	0.01
	Ref.Noise	0.14	0.09	0.01	**0.76**
normGLR	Ref.Rockfall	**0.91**	0.01	0.00	0.08
	Ref.Slide Quake	0.06	**0.88**	0.00	0.06
	Ref.EQ	0.01	0.01	**0.97**	0.01
	Ref.Noise	0.11	0.08	0.01	**0.80**
GTV	Ref.Rockfall	**0.92**	0.01	0.00	0.07
	Ref.Slide Quake	0.08	**0.87**	0.01	0.04
	Ref.EQ	0.03	0.01	**0.96**	0.01
	Ref.Noise	0.15	0.08	0.02	**0.75**

^*a*^ The predicted (Pred.) events are represented with respect to the events of the reference cataloged (Ref.), EQ stands for earthquake.

**Table 6:** Classification results of the cataloged events in [12] from continuous data ^*a*^.

	Pred.Rockfall	Pred.Slide Quake	Pred.EQ	Pred.Noise
Ref.Rockfall (65)	**60 (0.92)**	0	0	5
Ref.Slide Quake (18)	1	**15 (0.83)**	0	2
Ref.EQ (23)	0	0	**23 (1.00)**	0
Ref.Noise (13)	1	1	0	**11 (0.79)**

^*a*^ The predicted (Pred.) events are represented with respect to the events of the reference cataloged (Ref.). EQ stands for earthquake. The bold numbers represent the amount of correctly classified events and the sensitivity/recall of the corresponding class.

**Table 7:** Classification results of the additional manually expert-verified events from continuous data ^*a*^.

	Pred.Rockfall	Pred.Slide Quake	Pred.EQ	Pred.Noise	Pred.Others
Ref.Rockfall (71)	**60 (0.85)**	0	0	10	1
Ref.Slide Quake (21)	1	**15 (0.71)**	0	4	1
Ref.EQ (25)	0	0	**24 (0.96)**	1	0
Ref.Noise (616)	32	2	65	**257 (0.42)**	260

^*a*^ The predicted (Pred.) events are represented with respect to the events of the reference cataloged (Ref.). EQ stands for earthquake. The bold numbers represent the amount of correctly classified events and the sensitivity/recall of the corresponding class.

## Data Availability

The raw Resif data can be downloaded at http://ws.resif.fr/fdsnws/dataselect/1/, accessed on 8 January 2022. Additionally, the description of the site we analysed can be found at https://seismology.resif.fr/networks/#/MT, accessed on 8 January 2022.

## Appendix A

**Table A1:** List of features drawn from the literature: temporal s(t), power signal p(t), envelope e(t), auto correlation function ac(t), spectral f(v), cepstrum domain ce(v), template rockfall r(t), slide quake q(t), earthquake ea(t), noise n(t), and envelope es(t) with (5–10 Hz), (10–50 Hz), (5–70 Hz), (50–100 Hz) and (5–100 Hz) passband. PMF refers to Probability Mass Function.

Parameter	Description
Temporal Feature	
T1	Duration [12,30,31,32]
T2	STD of e(t) [29,31,32,33,34]
T3	RMS between the decreasing signal and $J\left(t\right)=Y_{max}-\frac{Y_{max}}{N-t_{max}}t$ [12]
T4	Optimum point of separation [33]
T5–7	Max, Mean, Median of e(t) [12,29,33]
T8, 9	Rising Decreasing duration s(t) [32]
T10	Entropy of s(t)−∑PMF(s(t))logPMF(s(t)) [34,35,36,37]
T11	Zero Cross Rate of s(t) [29,34]
T12, 13, 16, 19	Max CrossCor (q(t), r(t), ea(t), n(t)) [33]
T14, 15	Skewness of p(t) & e(t)$\frac{1}{N}\sum_{i=1}^{N}{\left(\frac{x\left(t_{i}\right)-\bar{x}}{\sigma }\right)}^{3}$ [12,29,30,32,34]
T17, 18	Kurtosis of p(t) & e(t)$\frac{1}{N}\sum_{i=1}^{N}{\left(\frac{x\left(t_{i}\right)-\bar{x}}{\sigma }\right)}^{4}$ [12,29,30,32,34]
T20,21,25,26	2dNormCrossCorAb (q(t), r(t), ea(t), n(t)) [33]
T22	Rate of decay of e(t) [35]
T23	Ratio of Max and mean of e(t)$\frac{T_{5}}{T_{6}}$ [32]
T24	Ratio of Max and Median of e(t)$\frac{T_{5}}{T_{7}}$ [32]
T27,40,47,48	No.peaks 2dNormCrossCorAb (q(t),r(t),ea(t),n(t)) [33]
T28–T32	Energy of es(t)$\sum_{i=1}^{N}\mathrm{es}{\left(t_{i}\right)}^{2}$ [12,32]
T33-37	kurtosis of es(t) [12,29,30,32,34]
T38&39	Energy of (1:N/3) and (N/3:N) of ac(t)$\sum_{i=1}^{\frac{N}{3}}\mathrm{ac}{\left(t_{i}\right)}^{2}\;$ $\sum_{i=\frac{N}{3}}^{N}\mathrm{ac}{\left(t_{i}\right)}^{2}$ [12]
T40	Int-ratio of ac(t)$\frac{T_{38}}{T_{39}}$ [12,32]
T41	No. of peaks of ac(t)————[12,32]
T42	duration of ac(t)$\underset{t}{max}\left(\mathrm{ac}\left(t\right)<0.2\max\left(\mathrm{ac}\left(t\right)\right)\right)/\left(T_{1}\right)$ [12]
T43	Measure of location $\sum_{i=1}^{N}ip\left(t_{i}\right)$ [37]
T44	Measure of dispersion $\sqrt{\sum_{i=1}^{N}{\left(i-T_{43}\right)}^{2}p\left(t_{i}\right)}$ [37]
T45	Measure of asymmetry $\frac{1}{T_{44}^{3}}\sum_{i=1}^{N}{\left(i-T_{43}\right)}^{3}p\left(t_{i}\right)$ [37]
T46	Measure of concentration around single value $\frac{1}{T_{44}^{4}}\sum_{i=1}^{N}{\left(i-T_{43}\right)}^{4}p\left(t_{i}\right)$ [37]
T49–52	No.peaks 2dNormCrossCorReal (q(t), r(t), ea(t), n(t)) [33]
T53–56	2dCrossCorAb (q(t), r(t), ea(t), n(t)) [33]
T57–60	2dCrossCorReal (q(t), r(t), ea(t), n(t)) [33]
Spectral Features	
F1–2	Absolute value of mean, max f(v) [12,30,32]
F3–4	Central frequency of the 1st and 2nd quartile [12]
F5	Max envelop of f(v) [12,33]
F6	No. of peaks >0.75 bandwidth f(v) [12,32]
F7	Dominate frequency [12,30,33]
F8	Spectral centroid [12,32,33,34,37]
F9–10	Median and variance of the normDFT [12,29,32,33,34]
F11	No. peaks of the autocorrelation ac(f(v)) [12,32]
F12,13,14,16	Energy of ([0,1/4], [1/4,1/2], [1/2,3/4], [3/4,1])*Fs [12]
F15	No. of peaks f(v) [12]
F17	Kurtosis of the Max of all DFTs [12]
F18,19	Mean ratio between the max and the mean and median of all DFTs [12]
Spectral Features	
F20,30,31	No. peaks in the curve showing the temporal evolution of the DFTs max, mean and median [12]
F21	Gamma 1 $\frac{\sum_{i=1}^{N}v_{i}f{\left(v_{i}\right)}^{2}}{\sum_{i=1}^{N}f{\left(v_{i}\right)}^{2}}$ [32]
F22	Gamma 2 $\sqrt{\frac{\sum_{i=1}^{N}v_{i}^{2}f{\left(v_{i}\right)}^{2}}{\sum_{i=1}^{N}f{\left(v_{i}\right)}^{2}}}$ [32]
F23	Gamma 3 $\sqrt{\left|F21^{2}-F22^{2}\right|}$ [32]
F24	Mean frequency $\frac{\sum_{i=1}^{N}\mathrm{PSD}\left(v_{i}\right)v_{i}}{\sum_{i=1}^{N}\mathrm{PSD}\left(v_{i}\right)}$PSD is the power spectral density of f(v) [12]
F25	Frequency bandwidth $2\sqrt{\frac{\sum_{i=1}^{N}\mathrm{PSD}\left(v_{i}\right){v_{i}}^{2}}{\sum_{i=1}^{N}\mathrm{PSD}\left(v_{i}\right)}-F_{24}^{2}}$ [12]
F26	Minimal frequency $\underset{v}{min}\left(\mathrm{PSD}\left(v\right)<0.2max\left(\mathrm{PSD}\left(v\right)\right)\right)$ [12]
F27	Maximal frequency $\underset{v}{max}\left(\mathrm{PSD}\left(v\right)<0.2max\left(\mathrm{PSD}\left(v\right)\right)\right)$ [12]
F28	Gyration radius $\sqrt{\frac{m_{3}}{m_{2}}}$$m_{i}$ is the ith moment [12]
F29	Spectral centroid width $\sqrt{{F_{8}}^{2}-{F_{28}}^{2}}$ [12,34]
F32-33	Ratio F20:F30, ratio F20:F31 [12]
F34, 35	No. peaks in the curve of the temporal evolution of the DFTs central and max frequency [12]
F36, 37	Mean distance between the curves of the temporal evolution of the DFTs max and mean or median frequency [12]
F38–40	Mean distance between (the 1st quartile and the median, the 3rd quartile and the median and the 3rd quartile and the 1st quartile) of all DFTs w.r.t time [12]
F41–44	2dCrossCorAbDWT with q(t), r(t), ea(t), n(t) [33]
F45–48	2dCrossCorRealDWT with q(t), r(t), ea(t), n(t) [33]
Cepstrum Features	
C1	STD ce(v)std(ce(v)) [35]
C2	Skewness of ce(v)$\frac{1}{N}\sum_{i=1}^{N}{\left(\frac{\mathrm{ce}\left(v_{i}\right)-\bar{\mathrm{ce}}}{\sigma }\right)}^{3}$ [35]
C3	Kurtosis of ce(v)$\frac{1}{N}\sum_{i=1}^{N}{\left(\frac{\mathrm{ce}\left(v_{i}\right)-\bar{\mathrm{ce}}}{\sigma }\right)}^{4}$ [35]
C4	Max value of ce(v)max(ce(v)) [35]
C5	Cepstrum No. peaks (echo) ce(v)
Acoustics [11]	
A1	No. peaks Linear prediction filter coefficients
Polarity [12]	
P1	Max eigenvalue of the covariance matrix
P2–3	Incidence angle, Polarization azimuth
P4–5	Degree of linear, Plane polarization
